# A *de novo* transcriptome analysis shows that modulation of the JAK-STAT signaling pathway by salmonid alphavirus subtype 3 favors virus replication in macrophage/dendritic-like TO-cells

**DOI:** 10.1186/s12864-016-2739-6

**Published:** 2016-05-23

**Authors:** Cheng Xu, Øystein Evensen, Hetron Mweemba Munang’andu

**Affiliations:** Department of Basic Sciences and Aquatic Medicine, Section of Aquatic Medicine and Nutrition, Faculty of Veterinary Medicine and Biosciences, Norwegian University of Life Sciences, P.O Box 8146, Oslo, NO-0033 Dep Norway

## Abstract

**Background:**

The Janus kinase (Jak) and signaling transducer activator of transcription (Stat) pathway mediates the signaling of genes required for cellular development and homeostasis. To elucidate the effect of type I IFN on the Jak/stat pathway in salmonid alphavirus subtype 3 (SAV3) infected macrophage/dendritic like TO-cells derived from Atlantic salmon (*Salmo salar* L) headkidney leukocytes, we used a differential transcriptome analysis by RNA-seq and the Kyoto encyclopedia of genes and genomes (KEGGs) pathway analysis to generate a repertoire of *de novo* assembled genes from type I IFN treated and non-treated TO-cells infected with SAV3.

**Results:**

Concurrent SAV3 infection with type I IFN treatment of TO-cells suppressed SAV3 structural protein (SP) expression by 2log_10_ at 2 days post infection compared to SAV3 infection without IFN treatment which paved way to evaluating the impact of type I IFN on expression of Jak/stat pathway genes in SAV3 infected TO-cells. In the absence of type I IFN treatment, SAV3 downregulated several Jak/stat pathway genes that included type I and II receptor genes, Jak2, tyrosine kinase 2 (Tyk2), Stat3 and Stat5 pointing to possible failure to activate the Jak/stat signaling pathway and inhibition of signal transducers caused by SAV3 infection. Although the suppressor of cytokine signaling (SOCS) genes 1 and 3 were upregulated in the IFN treated cells, only SOCS3 was downregulated in the SAV3 infected cells which points to inhibition of SOCS3 by SAV3 infection in TO-cells.

**Conclusion:**

Data presented in this study shows that SAV3 infection downregulates several genes of the Jak/stat pathway, which could be an immune evasion strategy, used to block the transcription of antiviral genes that would interfere with SAV3 replication in TO-cells. Overall, we have shown that combining *de novo* assembly with pathway based transcriptome analyses provides a contextual approach to understanding the molecular networks of genes that form the Jak/stat pathway in TO-cells infected by SAV3.

## Background

The Jak/stat pathway has been shown to contribute positively to innate immune responses against viral infections from drosophila to vertebrates [1–3]. Viruses have evolved counter measures that block different components of the Jak/stat pathway to prevent production of antiviral compounds [3, 4]. While very little information is available for salmon pancreas disease virus (SPDV also referred to as salmon alphavirus - SAV) causing pancreas disease of Atlantic salmon (*Salmo salar* L) and trout (*Oncorhynchus mykiss)*, it has been shown that other alphaviruses like chikungunya virus (ChikV) inhibit the stimulation type I and II IFN by blocking Jak/stat signaling, likely through NSP2 [1, 3]. Lin et al. [5] showed that Japanese encephalitis virus (JEV) block IFN induced Jak/stat signaling through a protein tyrosine phosphatase mediated mechanism. As pointed out by Fujii et al. [6] that strategies of counteracting Jak/stat signaling vary among viruses which include suppressing the phosphorylation of the Jaks, inhibition of nuclear translocation of activated Stats, degradation of different Jak/stat components as well as inducing the expression of negative regulators such as the SOCS proteins by host cells. This shows that viruses alter the Jak/stat signaling at different stages of the pathway.

Janus kinase (Jak) and signaling transducer and activator of transcription (Stat) is an intracellular signaling pathway that mediates the effects of a large number of cytokines and growth factors [7]. It is a pleiotropic cascade used to transduce a network of signals for the development and homeostasis of cells from vertebrates to insects. Jak activation stimulates cell proliferation, differentiation, migration and apoptosis. In general, the Jak/stat pathway is based on a few principle components involving a variety of ligands and their receptors [8]. Intracellular activation of the Jaks takes places when ligand binding induces dimerization of receptor subunits on cell surfaces [8]. For signal transduction through dimerization, the cytoplasmic domain of the receptor subunits must be associated with Jak tyrosine kinases, which juxtaposes the Jaks to allow for their transphosphorylation. The transphophorylated Jaks phosphorylate additional targets that include the Stats. Stats are latent transcriptional factors found in the cytoplasm in an inactive state until they are activated. Phosphorylated Stats migrate to the nucleus where they modulate the transcription of different target genes. In general, the Jak/stat pathway provides a direct mechanism in which extracellular signals are translated into transcriptional responses that modulate the expression of different target genes.

Apart from Stats, other effector molecules that contribute to Jak/stat signaling include the signal-transducing adaptor molecules (STAMs) that facilitate the transcriptional activity of specific target genes such as transcriptional regulator Myc (Myc) [9] and the stat-interacting protein (StlP) which serves as a scaffold to phosphorylate the Stats by the Jaks [8]. On the other hand, three major classes of negative regulators have been identified which include the suppressor of cytokine signaling (SOCS), protein inhibitors of activated stats (PIAS) and protein tyrosine phosphates (PTPs) [10]. The SOCS family serves as a negative feedback loop on the Jak/stat circuit in which activated Stats stimulate the transcription of SOCS genes that bind to phosphorylated Jaks and their receptors to turn off the signaling pathway [11, 12]. The PTPs includes the SHP-2 phosphatases, which has two SH2 (src-homology-2) domains that bind to the Jaks to initiate the dephosphorylation of the Jak and Stat proteins [13]. The PIAS proteins bind to activated Stat dimers to prevent their signal transducing activities although the exact mechanism in which this is carried out has not been established [13, 14]. Put together, the Jak/stat cascade is a tightly controlled pathway regulated by different effector proteins and negative regulators that prevent overstimulation of the paracrine pathway.

Therefore, to identify genes that alter Jak/stat signaling induced by viral infections calls for a comprehensive profiling of genes induced by virus replication during infection progression in host cells. In the present study we used RNA-seq and put together a *de novo* assembly of genes belonging to the Jak/stat pathway induced by salmonid alphavirus subtype-3 (SAV-3) infection in TO-cells. TO-cells are a continuous cell-line derived from Atlantic salmon headkidney leukocytes characterized to possess dendritic cell-like properties [15]. SAV3 is a potent inducer of type I IFN responses in TO-cells while at the same time, the virus replicates vividly under high IFN I levels [15–17]. Pretreatment of permissive cells with recombinant salmon IFN I will protect the cells against CPE but the effect of this treatment will depend on time of treatment relative to infection [17]. The mechanisms employed by SAV3 to circumvent or block the antiviral responses of the host cells are not known. Hence, in this study we wanted to identify the profile of genes linked to the Jak/stat pathway induced by SAV3 infection in TO-cells and to compare their expression patterns in cells simultaneously infected with SAV3 and treated with type I IFN to SAV3-only infected cells. The aim was to understand any modulation of the antiviral responses elicited by SAV3 infection compared to partly protected (IFN I/SAV3) cells. To do this, we used the Kyoto encyclopedia of genes and genomes (KEGG) database analysis to generate pathways of differentially expressed genes generated by *de novo* assembly using RNA-seq from SAV3 infected TO-cells. In general, we anticipate that data presented here shall serve as a roadmap to elucidating the cellular networks linked to the Jak/stat pathway used by SAV3 to evade the host defensins in infected fish.

## Methods

### Cell culture, virus infection and IFN treatment

TO-cells derived from Atlantic salmon (*Salmo salar* L) head kidney leukocytes characterized to possess macrophage/dendritic-like properties were use in this study [15, 16]. The TO-cells, were propagated in HMEM (Eagle’s minimal essential medium [MEM] with Hanks’ balanced salt solution [BSS]) supplemented with L-glutamine, MEM nonessential amino acids, gentamicin sulfate, 10 % FBS and were incubated at 20 °C. The Salmon alphavirus subtype 3 (Genebank accession JQ799139) used to inoculate the TO-cells has previously been described [18]. One batch of TO-cells was treated with 500 ng/ml of Atlantic salmon Type I IFN in triplicates and was simultaneously infected with SAV3. Another batch of TO-cells was only infected with SAV-3, without type I IFN treatment. The virus was inoculated at an MOI 1 when the cells were 80 % confluent in both groups. Thereafter, both the SAV-3 infected cells with and without IFN treatment were incubated at 15 °C in maintenance media using HMEM growth media supplemented with 2 % FBS. The mock group was only treated with maintenance media without SAV3 infection and no type I IFN treatment. Cells were harvested after 48 h and were used for RNA extraction used for RNA-seq analysis. Another set of three independent replicates subjected to the same treatment was used for qRT-PCR analysis. All studies in TO-cells were carried out in triplicates.

### RNA isolation

Extraction of total RNA was carried out using the RNeasy mini Kit with on-column DNase treatment according to the manufacturer’s instructions (Qiagen, Hilden, Germany). The concentration and quality of RNA was analyzed using a Nanodrop ND1000 (Nanodrop Technologies, Wilmington, USA) and Agilent 2100 Bioanalyzer (Agilent Technologies, USA), respectively.

### Library construction, sequencing and data analysis for RNA-Seq

Triplicates of equal quantities of total RNA from IFN-treated and non-treated-TO-cells infected with SAV3 together with Mock cells were mixed to prepare the pooled RNA sample for each group. The pooled samples were treated with DNase I for the degradation of any possible DNA contamination followed by enrichment using oligo(dT) magnetic beads. Thereafter, the mRNA was fragmented into short fragments of approximately 200 bp by mixing with the fragmentation buffer. This was followed by first strand cDNA synthesis using random hexamer-primer, which was followed by adding a buffer containing dNTPs, RNase H and DNA polymerase I to synthesize the second strand. The generated double strand cDNA was then purified with magnetic beads and the end reparation and 3’-end single nucleotide A (adenine) addition was then performed. Thereafter, sequence adaptors were ligated to the fragments, which were later enriched by PCR amplification. The Agilent 2100 Bioanaylzer and ABI StepOnePlus Real-Time PCR System (Biorad.com) were used to qualify and quantify the sample library during quality check (QC step). After the QC step, library products were ready for RNA-sequencing using Illumina HiSeqTM 2000, BGI-Hong Kong. For RNA-seq analysis, clean reads were obtained after removal of adaptor sequences, reads having greater than 10 % unknown bases as well as removal of low quality bases (base with quality value ≤ 5) greater than 50 % in a read.

### Functional annotation and gene ontology classification

Raw reads generated by illumina sequencing were used to generate a library of clean reads after quality check, which was used for transcriptome *de novo* assembly using the Trinity software (http://trinityrnaseq.github.io [19]. Thereafter, assembled unigenes were used for annotation for the classification of gene functions by searching different protein databasesusing BlastX version 2.2.23 as previously described [18]. Data from BlastX was used to extract the coding regions (CDS) from unigene sequences and translate them into peptide sequences. As for unigenes without hits in BlastX, the ESTScan was used to predict their CDS and to decide their sequence direction. Unigenes having NR annotation were further analyzed with Blast2go (https://www.blast2go.com/) to obtain their gene ontology (GO) annotations for classification according to GO functions using the Web Gene Ontology (WEGO) annotation software.

### Identification of differentially expressed genes and KEGG pathway analysis

Identification of differentially expressed genes (DEGs) was carried out as previously described [18]. In this study, DEGs were generated based on comparing the RPKM mapped reads from TO cells treated with type I IFN simultaneously infected with SAV3 versus mock TO-cells that were designated as TO-VS-IFN/SAV3, while the second comparison was based on TO-cell infected with SAV-3 only, without IFN treatment, versus mock TO-cells that were designated as TO-VS-SAV3. Only genes with a threshold of false discovery rate (FDR) <0.001 and an absolute value log_2_ratio > 1 were considered as differentially expressed. Thereafter, all DEGs were assigned KEGG orthologue (KO) identifiers which were later used for pathway analysis using the KEGG database (http://www.genome.jp/kegg/). In addition, we used the TIGR gene indices clustering tools (TGICL) (http://www.tigr.org/tdb/tgi) [19, 20] software to remove redundancy.

### Validation of RNA-Seq Data by qRT-PCR

To confirm the validity of the DEGs generated by RNA-Seq, 13 DEGs obtained from the TO-VS-IFN/SAV3 and TO-VS-SAV3 were randomly selected for the qRT-PCR validation test. Gene expression of three independent replicates of TO-cells simultaneously treated with type I IFN and infected with SAV-3 (TO-VS-IFN/SAV3) was compared with none-type I IFN treated cells infected with SAV3 (TO-VS-SAV3) relative to gene expression of untreated TO-cells before normalized to β-actin. Cells used for independent replicate biological testing were independent of the cells used for RNA-seq data analysis. The qRT-PCR was carried out as previously described [18] using 100 ng total RNA as a template for each gene in a master-Mix final volume of 25 μl according to manufacturer’s recommendations (Roche Diagnostics, Mannheim, Germany). Primers used in the study are shown in Table 1 and the primer-master-mix solutions were first incubated for reverse transcription at 50 °C for 10 min followed by PCR initial activation at 95 °C for 5 min and 40 amplification cycles (10 s at 95 °C and 30 s at 60 °C). The specificity of each PCR products was confirmed by melting-curve analysis and agarose gel electrophoresis. The delta C_t_ method was used to calculate the fold-increase in gene expression relative to the β-actin control group [21]. All quantifications were normalized to β-actin control group. The qRT-PCR was done thrice, with each repeat study having three independent replicates of IFN treated (TO-VS-IFN/SAV3) and non-treated cells (TO-VS-SAV3). The mean of each gene from the three independent replicates after three repeats was used to compute the correlation coefficient test between RNA-seq and qRT-PCR data.

**Table 1:** Primer sequencing for qRT-PCR

Primer name	Sequence	GeneBank accession no
SAV-3 E2-F	CAGTGAAATTCGATAAGAAGTGCAA	EF675594
SAV-3 E2-R	TGGGAGTCGCTGGTAAAGGT	
β-actin-F	CCAGTCCTGCTCACTGAGGC	AF012125
β-actin-R	GGTCTCAAACATGATCTGGGTCA	
STAT1-F	CGGGCCCTGTCACTGTTC	GQ325309
STAT1-R	GGCATACAGGGCTGTCTCT	
STAT2-F	AAAACCCCCTCCTCTTCCTC	NM_001145424
STAT2-R	TTAGCCTCCAGTGTGTTCC	
IFNGR1-F	TCAAACACTTCCCTCTACCAC	EU244876
IFNGR1-R	GTATTCTGTCCAACAGCAGTC	
IFNGR2-F	AGTCCATCCAAGAGTCAGAAAG	EU244877
IFNGR2-R	CCACTACCAAGACACCCAAC	
IL-10RB-F	ATCAGTCAGTCTCGACTCGC	BT047745
IL-10RB-R	TCACCCATATCTTCCCCATCC	
IL-20RA-F	TTCAACCTGCTCTCAGTCC	NM_001141080
IL-20RA-R	GCCTTCATCATCATCTTCTTCC	
IL-15RA-F	TGTGTCTCCAACAGAACCCC	NM_001124572
IL-15RA-R	TTACTACCACCCCACCTACACC	
IL-13RA-F	GCTTTTCCCCACGATACCAC	NM_001140169
IL-13RA-R	ATTCCACCTCCTCCTCAACC	
LIFRA-F	TTTAACCTATCCTGGTCCGC	HM190263
LIFRA-R	TACCTTCACCCACTCCACTC	
GHR1-F	CGCACCAACACAGATAATGAG	NM_001123576
GHR1-R	GCAAAGACAAGCAGGACAG	
IRF9-F	ACAAAGTTTACTGCCTTGTACC	NM_001173719
IRF9-R	ACAACCTCCTCCTCCTTCAC	
Myc-2-F	TCCGCTTCCTACCACAAAC	NM_001173816
Myc-2-R	TCCTCCTCATCCTCCACATC	
AKT2-F	TTACACTGACACCCTACTGAC	NM_001139690
AKT2-R	AGCCATCCTTCCCTGACTAC	
Bcl-X-F	TTGAACCTTGCATCCCACC	NM_001141086
Bcl-X-R	AGTCCTAGATACCTCCCTCC	

### Virus quantification

In order to determine the effect of type I IFN treatment on SAV3 replication in TO-cells, virus quantification was determined after 48 hrs replication at 15 °C for the IFNα treated and non-treated groups using a guided sequence format for the structural proteins (SPs) and non-structural proteins (NSPs) of SAV3 (GeneBank accession nos. AFJ15605.1 and AF15604.1) based on the RNA-seq data. The Fragment per kilobase per million (FPRM) method was used to read the number of fragments of NSPs and SPs expressed by SAV3 after 48 hrs replication at 15 °C in the IFNα treated and non-treated cells. In order to verify the validity of the virus quantification data generated by RNA-Seq, quantitative RT-PCR (qRT-PCR) was used to determine the SAV3 replication level in the IFNα treated and non-treated cells using the E2 SP expressed during SAV3 replication. The primer sequences used for E2 quantification are shown in Table 1 and the qRT-PCR method used has previously been described by Xu et al. [17].

## Result

### Virus quantification

**Fig. 1:**
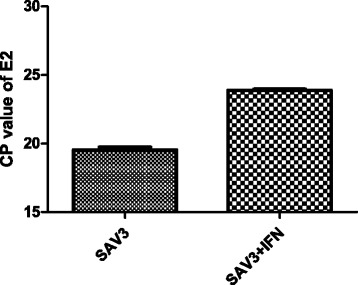
Shows virus titration based on detection of the E2 structural protein for SAV3 by the ΔΔC*p*-value analytical method at 48 hours after inoculation in TO-cells. Simultaneous treatment of IFNα and SAV3 infection (TO-VS-IFN/SAV3) reduced the replication of the virus by 20 fold relative to SAV3 infected TO-cells (TO-VS-SAV3) without IFNα treatment

First we documented the effect of IFN I treatment on the SAV3 SP (structural protein) expression. We found that the non-IFNα treated cells had 10^2^ higher expression levels of SAV3 SPs than the IFN I treated cells (Table 2). This shows that simultaneous treatment with IFN I reduced the level of synthesis of mRNA SPs from subgenomic viral RNA. Similarly, the NSP mRNAs (non-structural proteins mRNAs) expressed in the non-IFNα treated cells were 10^0.8^ higher than in the IFNα treated group (IFN/SAV3). The log2 ratio of the IFNplusSAV3/SAV3 for the SPs and NSPs showed a downregulation of -7.4092 and -2.3219, respectively, showing that type I IFN added concurrently with SAV-3 infection significantly reduced virus replication in TO cells (Table 2). Consistent with RNA-seq data shown in Table 2, Fig. 1 shows a 20 fold-reduction in SAV3 replication in the IFN I treated cells (IFN/SAV3) relative to the untreated cells based on the E2 SPs detected by qPCR using the Cp-value test. Thus, qPCR confirms the validity of our RNA-seq data analysis by showing that a downregulation of SAV3 SPs in IFN I treated cells (IFN/SAV3) versus SAV3 infected cells only.

**Table 2:** Comparison structural and non-structural protein expression in IFN I treated and non-treated cells 48 hours post SAV3 infection

Parameters	Structural Protein (SP)	Non-structural protein (NSP)
Gene ID	AFJ15605.1	AFJ15604.1
Gene length	3960	7764
Expression of SAV3	1800842.81	62.71
Expression of SAV3 (log10)	1 × 10^6.26^	1 × 10^1.80^
Expression of IFN/SAV3	9403.57	11.43
Expression of IFN/SAV3 (log10)	1 × 10^3.97^	1 × 10^1.06^
SAV3-FPKM	42259.47	0.75
IFN/SAV3-FPKM	248.61	0.15
log2 Ratio (IFN/SAV3/SAV3)	-7.41	-2.32
Up/Down-Regulation	Down	Down
*P*-value	0	1.33E-08
FDR	0	1.14E-07

### Functional annotation based on the KEGG pathway analysis

Based on data generated by illumina sequencing, we constructed three libraries that yielded a total of 23 331 788, 24 608 338 and 23 421 631 raw reads for the IFN-treated and non-IFN treated TO-cells infected with SAV3 together with mock TO-cells, respectively. The total unique match in total mapped reads was >90 % after removal of redundancy using the TGICL software (http://www.tigr.org/tdb/tgi) suggesting low redundancy. After quality check for clean reads and filtration [18], 20,115 unigenes from the TO-VS-IFN/SAV3 and TO-SV-SAV3 groups assigned to the KEGG orthologues (KO) were identified using the KEGG database (http://www.genome.ad.jp/kegg/), which has been widely used to generate pathway networks for model organisms such as the Zebra fish (*Danio rerio*) [22, 23] and mouse (*Mus musculus*) [24] as well as several non-model organisms [25, 26]. The profile of genes identified using KO-based on the RPKM method in which only genes with an FDR < 0.001 and an absolute value log_2_ratio > 1 were considered differentially expressed showed that the TO-VS-IFN/SAV3 had 1629 DEGs whose KOs were mapped to 237 pathways of which 49 DEGs belonged the Jak/stat pathway (Table 3). On the other hand, the TO-VS-SAV3 had a total of 9135 DEGs assigned to KO-identifiers, which was 7506 DEGs more than the TO-VS-IFN/SAV3. As a result, the TO-VS-SAV3 had 252 pathways identified by the KEGG database being 15 pathways more than those identified in the TO-VS-IFN/SAV3 (Table 3). In terms of Jak/stat genes, the TO-VS-SAV3 had a total of 135 DEGs expressed being 86 DEGs more than the TO-VS-IFN/SAV3. In general, the TO-VS-SAV3 had more DEGs and pathways identified using the KEGG database analysis than the TO-VS-IFN/SAV3 although the TO-VS-IFN/SAV3 had a higher enrichment Qvalue (1.136E-06, *p* value = 8.07E-08) than the TO-VS-SAV3 (5.899E-01, *p* value = 8.07E-01) (Table 3).

**Table 3:** Comparison of TO-VS-IFN/SAV3 and TO-VS-SAV3

Parameters	TO-VS-IFN/SAV3	TO-VS-SAV3
KEGG orthology unigenes (KO)	20,115	20,115
Differentially expressed genes (DEGs)	1629	9,135
Total JAKSTAT DEGs	49	135
Upregulated DEGs	40	16
Down regulated DEGs	9	119
Number of KEGG pathways	237	252
JAKSTAT pathway Qvalue	1.14E-06	5.90E-01
JAKSTAT pathway *p*-value	8.07E-08	8.07E-01

**Fig. 2:**
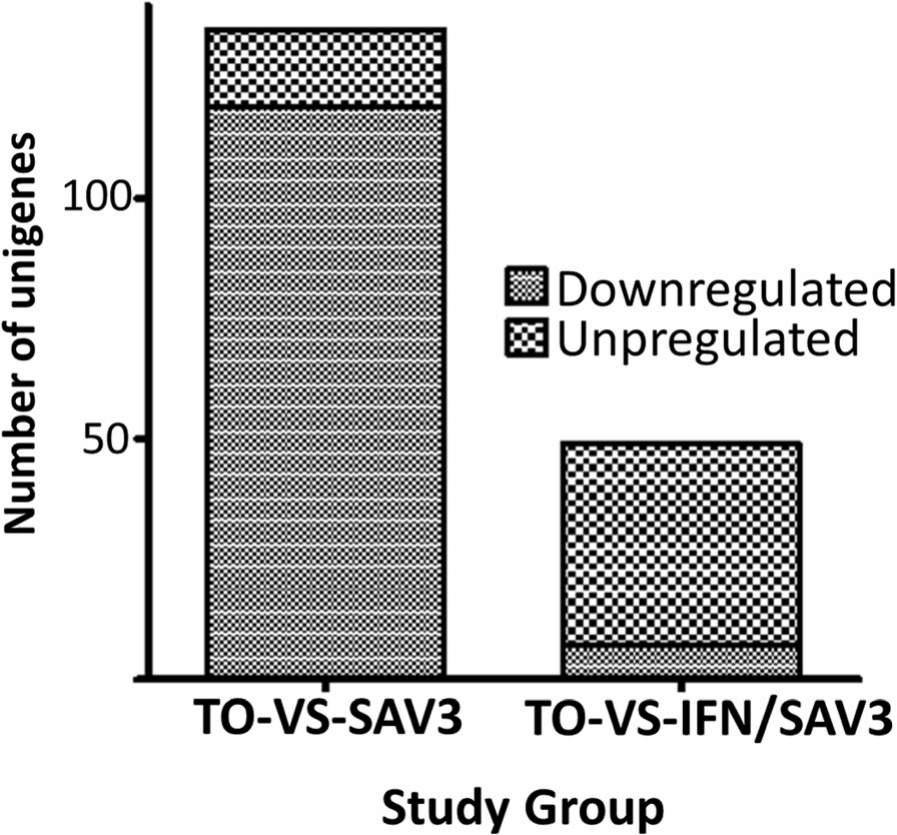
Comparison of downregulated and upregulated differentially expressed genes (DEGs) induced in TO-cells infected by SAV3 without type I FN treatment (TO-VS-SAV3) and type I IFN treated cells infected with SAV3 (TO-VS-IFN/SAV3)

To gain more insights on the impact of type I IFN treatment on the Jak/stat pathway in TO-cells infected with SAV-3, we compared the DEGs expressed from the TO-VS-IFN/SAV3 with DEGs from the TO-VS-SAV3. Figure 2 shows that of the 135 DEGs expressed in the TO-VS-SAV3, 119 DEGs were downregulated while 16 were upregulated. In the TO-VS-IFN/SAV3 group 40 DEGs were upregulated while 9 DEGs were downregulated. Put together, these findings show that the proportion of upregulated DEGs in the TO-VS-IFN/SAV3 (81.6 %, *n* = 49) was higher than in the TO-SV-SAV3 group (11.9 %, *n* = 135) while the proportion of downregulated DEGs for the TO-SV-SAV3 (88.1 %, *n* = 135) was higher than the TO-VS-IFN/SAV3 group (18.4 %, *n* = 49). This is strongly suggesting that type I IFNs upregulate several genes of the Jak/stat pathway that were downregulated by SAV3 infection alone in TO-cells. Hence, to further elucidate the type of genes suppressed by SAV3 infection in the absence of type I IFN treatment, we broadly classified the DEGs in the Jak/stat pathways for both the TO-VS-IFN/SAV3 and TO-VS-SAV3 into type I and II receptor genes, Jaks, Stats and negative regulator genes as shown below.

### Type I and II receptor genes

**Fig. 3:**
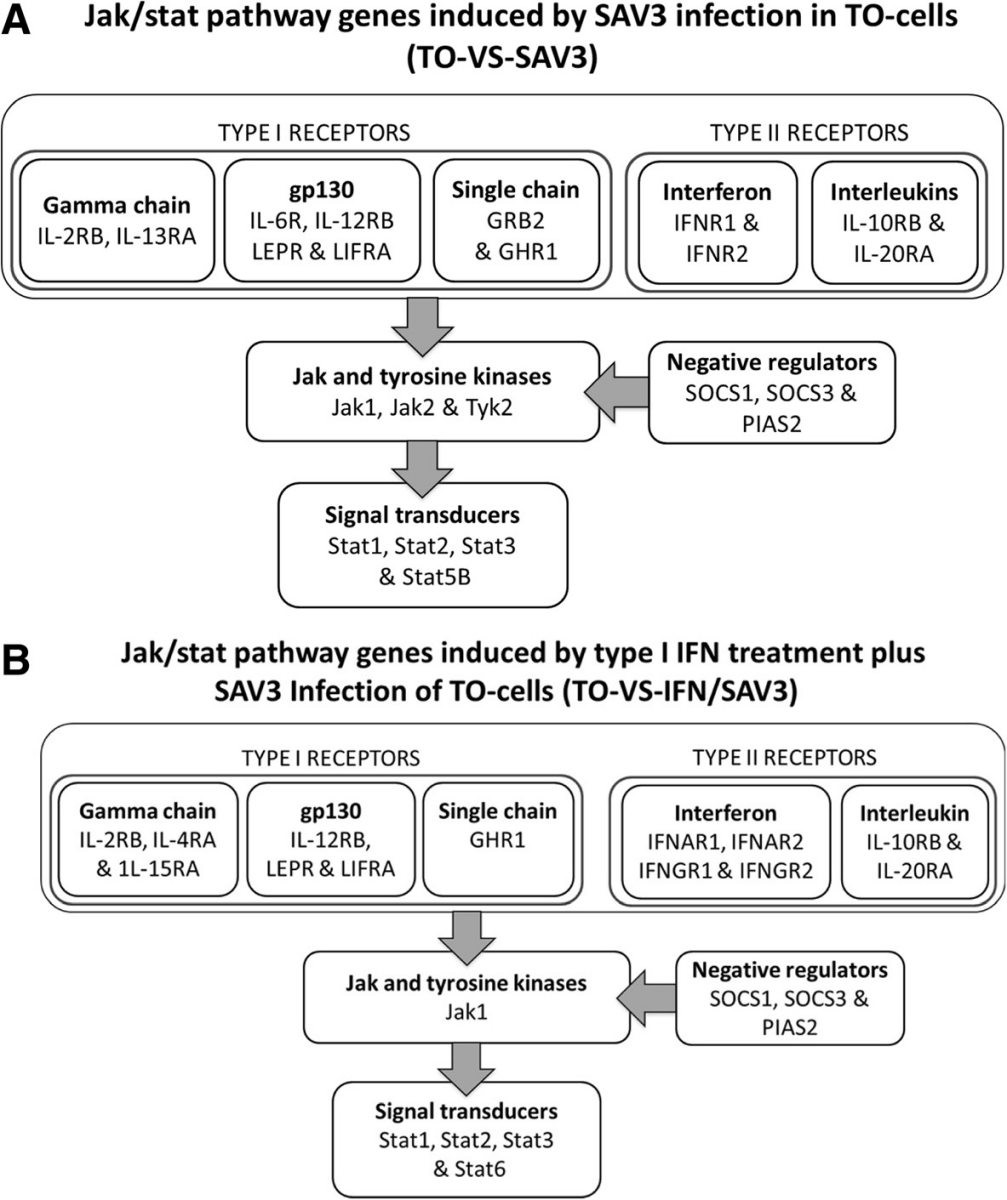
Schematic presentation of the Jak/stat pathway genes induced by SAV-3 infection without type I IFN treatment (TO-VS-SAV3) and type I IFN treated cells (TO-VS-IFN/SAV3) infected with SAV3. Note each group is subdivided into type I and II receptors, Jak and tyrosine kinases, negative regulators and signal transducers. **a** shows that the TO-VS-SAV3 had more type I receptor than the TO-VS-IFN/SAV3 shown in **b**. Among the type II receptors, the IFNGR1 and IFNGR2 were only reported in the TO-VS-IFN/SAV3 and not in the TO-VS-SAV3 while among the Jaks, Tyk2 was only expressed in the TO-VS-SAV3 and not in TO-VS-IFN/SAV3. For the signal transducer, Stat5B was only expressed in the TO-VS-SAV3 and not in the TO-VS-IFN/SAV3 while Stat6 was only expressed in the TO-VS-IFN/SAV3 and not in the TO-VS-SAV3

Differentially expressed genes belonging to receptor ligands that activate the Jak/stat pathway were classified into type I and II receptor-genes. As shown in Fig. 2 and Table 3, the type I receptor family was subdivided into the gamma chain (γC), gp130 and single chain (homodimer) receptor genes while the type II receptor family comprised of type I and II IFN receptor genes. The major difference between the TO-VS-IFN/SAV3 and TO-VS-SAV3 observed is that interleukin (IL) 13RA, IL-6RA, and Growth factor receptor-bound protein 2 (GRB2) were only downregulated in the TO-VS-SAV3 while IL-4RA and IL-15R were only upregulated in the TO-VS-IFN/SAV3. In general, there were more type I receptor genes differentially expressed in the TO-VS-SAV3 than in the TO-VS-IFN/SAV3 (Fig. 3) of which most of them were downregulated (Table 4).

**Table 4:** Type I and II receptor genes

Gene name	Abbr	Unigene	NCBI	KEGG	TO-VS-SAV3			TO-VS-SAV+IFN		
					Log2 (Fold change)	Reg	*P*-value	Log2 (Fold change)	Reg	*P*-value
Type I cytokine and noncytokine receptors										
(i) Gamma chain (_Y_C) family										
Interleukin-2 receptor beta chain	IL-2RB	Unig32859	NP_001134020.1	K05069	6.56	Up	1.52e-02	9.68	Up	3.89e-16
Interleukin-4 receptor alpha chain	IL-4RA	CL1700.1	NP_001133565.1	K05071				1.04	Up	4.17e-24
Interleukin-13 receptor alpha chain	IL-13RA	Unig8578	NP_001133641.1	K05076	-1.89	Down	1.06e-71			
Interleukin-15 receptor alpha chain	IL-15RA	Unig15594	NP_001118044.1	K05074				1.29	Up	5.72e-09
(ii) gp130 family										
Interleukin-6 signal transducer GP130	IL-6RA	Unig10219	CBL94873.1	K05060	-1.05	Down	7.91e-05			
Interleukin-12 receptor beta chain	IL-12RB	CL7886.1	XP_003452899.1	K05064	-1.91	Down	6.32e-52	-1.35	Down	2.74e-22
Leptin receptor	LEPR	CL10577.1	NP_001158237.1	K05062	-2.57	Down	2.00e-5	-1.77	Down	5.00e-4
Leukemia inhibitory factor receptor alpha	LIFRA	Unig7438	ADV36655.1	K05080	-1.92	Down	5.58e-168	-1.36	Down	4.60e-94
(iii) Single chain family										
Growth hormone receptor isoform 1	GHR1	Unig15500	NP_001117048.1	K05080	-2.17	Down	5.74e-27	-1.05	Down	4.78e-08
Growth factor receptor-bound protein 2	GRB2	Unig8178	XP_003455002.1	K04364	-1.16	Down	3.05e-25			
Type II cytokine receptors										
(i) IFN family receptor										
Interferon receptor 1	IFNAR1	Unig34816		K05130	1.86	Up	3.24e-03	3.47	Up	9.38e-08
Interferon receptor 2	IFNAR2	Unig11244		K05131	1.23	Up	2.97e-11	2.58	Up	1.07e-65
Interferon-gamma receptor 1	IFNGR1	CL9561.2	ABY87188.1	K05132				1.14	Up	1.55e-124
Interferon-gamma receptor 2	IFNGR2	CL3893.1	ABY87189.1	K05133				1.57	Up	2.44e-140
(ii) Interleukin receptors										
Interleukin-10 receptor beta chain	IL-10RB	Unig6317	ACI67546.1	K05135	3.78	Up	2.11e-04	4.92	Up	4.2e-05
Interleukin-20 receptor alpha chain	IL-20RA	Unig7932	NP_001134552.1	K05136	5.31	Up	8.93e-66	7.33	Up	1.31e-280

**Fig. 4:**
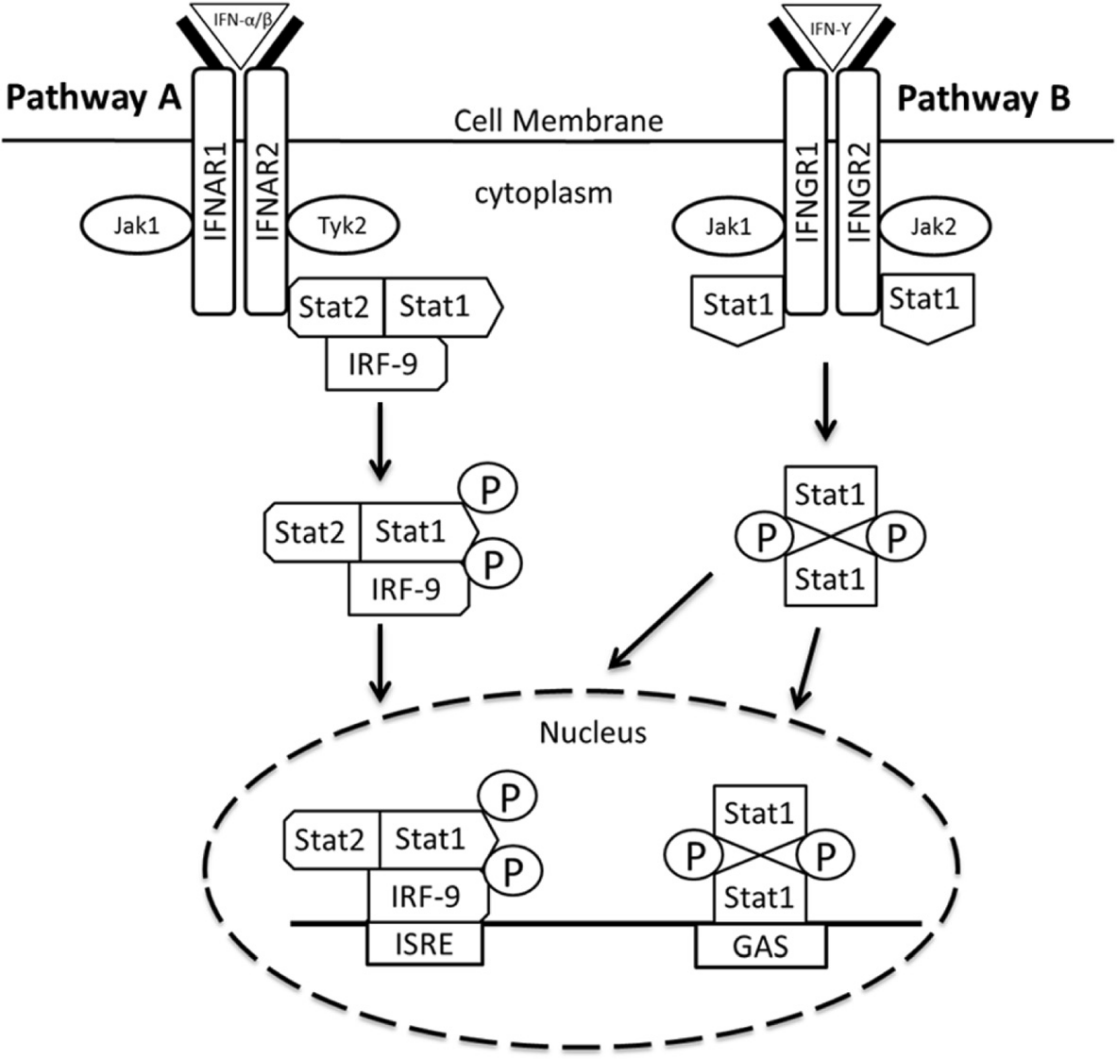
Shows the schematic presentation of the IFN-α/β and IFNγ receptors linked to the Jaks for the TO-VS-IFN/SAV3 and TO-VS-SAV3. In pathway A, the IFN-α/β binds Jak1 and Tyk2 receptor subunits linked to Stats 1 and 2, followed by a pathway that translocates the phophorylated Stats into nucleus. In pathway B, the IFNγ receptor is shown to bind to Jaks 1 and 2, which are linked to Stat1 and the phosphorylated Stats are shown to translocate to the nucleus. Based on data shown in Table 2, the TO-VS-IFN/SAV3 uses both pathways A and B using the IFN-α/β and IFNγ ligands while the TO-VS-SAV3 only uses pathway A using the IFN-α/β ligand. This model is adapted from IFN-receptor signaling pathways for the Jak/stat pathway in higher vertebrates [41, 42, 70]

The type II receptor genes were subdivided into two groups namely the interleukin (IL) and IFN receptor genes. As shown in Table 3, the IL-receptor genes comprised of IL-10RB and IL-20RA of which both were upregulated in the TO-VS-IFN/SAV3 and TO-VS-SAV3. The IFN-receptor genes comprised of IFN-α/β and IFNγ receptor genes (Table 4). The IFN-α/β receptor genes comprised of IFNR1 and IFNR2 which were upregulated in both the TO-VS-SAV3 and TO-VS-IFN/SAV3 while the IFNγ receptor genes comprised of IFNGR1 and IFNGR2 that were only expressed in the TO-VS-IFN/SAV3 in which both were upregulated (Table 4). Fig. 4 shows a schematic layout of the common IFN-receptor signaling pathways based on mammalian model species [8, 27] of which applying data presented in Table 3 shows that the IFN-receptor-genes expressed by the TO-VS-IFN/SAV3 were linked to both pathways A and B while the IFN-receptor-genes expressed by the TO-VS-SAV3 was only linked to pathway A.

### Janus and tyrosine kinase genes

**Fig. 5:**
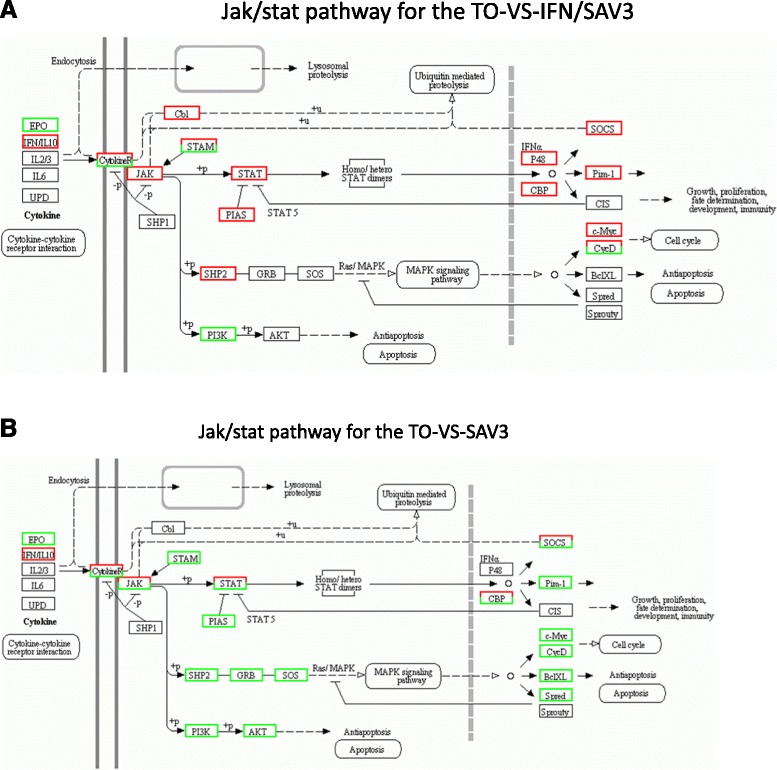
Shows Jak/stat pathway analysis for the TO-VS-IFN/SAV3 and TO-VS-SAV3 using the KEGG pathway software analysis. **a** shows the Jak/stat network pathway for the TO-VS-IFN/SAV3 while **b** shows the Jak/statcnetwork pathway of the TO-VS-SAV3. Red square depicts upregulated genes while the green square show the downregulated genes in the pathway. A mixed shading of red/green squares represents a mixed population of upregulated and downregulated genes. Note that there were more downregulated genes in the TO-VS-SAV3 (Fig. 5A) than in the TO-VS-IFN/SAV3 (Fig. 5B)

Three genes belonging to the Jaks were differentially expressed namely Jak1, Jak2 and tyrosine kinase (Tyk2) (Fig. 3, Table 5). All three Jaks were differentially expressed in the TO-VS-SAV3 while only Jak1 was differentially expressed in the TO-VS-IFN/SAV3. Jak1 was upregulated in both the TO-VS-SAV3 and TO-VS-IFN/SAV3 while Jak2 and Tyk2 were downregulated in the TO-VS-SAV3 (Table 5). Network pathways linking the Jaks to other genes in the Jak/stat pathway are shown in Fig. 5A for the TO-VS-IFN/SAV3 and in Fig. 4B for TO-VS-SAV3. Note that Fig. 5A shows upregulation of all Jaks being in conformity with observations made in Table 5 in which only Jak1 was differentially expressed in the TO-VS-IFN/SAV3 and it was upregulated. On the other hand, Fig. 5B shows a mixed representation of upregulated and downregulated Jak-genes, which is in agreement with observations shown in Table 4 in which Jak1 was upregulated whiles Jak2 and Tyk2 were downregulated.

**Table 5:** Repertoire of Jak kinuses signal transducer and activator of transcription, and negative regulatory genes

Gene name	Abbr	Unigene	NCBI	KEGG	TO-VS-SAV3			TO-VS- IFN/SAV3		
					Log2 (Fold change)	Reg	*P*-value	Log2(Fold Change)	Reg	*P*-value
A: Jaks										
Tyrosine-protein kinase JAK1	JAK1	CL2001.7	NP_571148	K11217	1.17	Up	1.87e-185	2.61	Up	1.01e-05
Janus kinase 2	JAK2	Unig38863	ACU12481	K04447	-1.97	Down	3.84e-05			
Tyrosine kinase 2	TyK2	Unig6068	ACU12483.1	K11219	-1.07	Down	7.70e-27			
B: Signal transducers										
Signal transducer and activator of transcription 1	STAT1	CL2066.1	NP_001134757.1	K11220	6.03	Up	3.06e-68	7.11	Up	1.20e-4
Signal transducer and activator of transcription 2	STAT2	Unig6362	NP_001138896.1	K11221	1.88	Up	1.31e-06	2.67	Up	1.10e-4
Signal transducer and activator of transcription 3	STAT3	CL10061.2	GU933429.1	K04692	-1.32	Down	4.00e-05			
Signal transducer and activator of transcription 5B	STAT5	Unig39041	XP_003454096.1	K11224	-2.32	Down	3.00e-05			
Signal transducer and activator of transcription 6	STAT6	Unig2573	ACU12488.1	K11225				1.29	Up	3.83e-57
C: Negative loop feedback										
Suppressor of cytokine signaling 3	SOCS3	Unig14487	NP_001139640.1	K04696	-1.25	Down	1.02e-07	1.89	Up	1.04e-07
Suppressor of cytokine signaling 1	SOCS1	Unig8629	CCC15083.1	K04694	6.86	Up	1.31e-207	7.78	Up	1.11e-04
E3 SUMO-protein ligase PIAS2	PIAS2	Unig18900	XP_003457088.1	K16063	-1.06	Down	2.00e-05	1.01	Up	7.71e-07

### Signal transducer and activator of transcription genes

Five Stat genes were differentially expressed in this study namely Stats 1, 2, 3, 5B and 6 (Table 5). As shown in Fig. 2A, Stats 1, 2, 3 and 5B were differentially expressed in the TO-VS-SAV3 of which Stats 1 and 2 were upregulated while Stats 3 and 5B were downregulated (Table 5). In the TO-VS-IFN/SAV3, Stats 1, 2 and 6 were upregulated (Table 5, Fig. 3B). The network links of the Stats to other genes in the Jak/stat pathway for the TO-VS-SAV3 and TO-VS-IFN/SAV3 are shown in Fig. 5A and B, respectively. Note that all Stats in the TO-VS-IFN/SAV3 were upregulated in Fig. 5A conforming to observations shown in Table 5 in which all Stats were upregulated while Fig. 5B shows a mixed representation of upregulated and downregulated Stats for the TO-VS-SAV3 being in line with observation shown in Table 4 in which Stats 1 and 2 were upregulated while Stats 3 and 5B were downregulated.

### Negative regulatory genes

The negative regulatory genes for the Jak/stat pathway differentially expressed in this study were classified into the suppressor of cytokine signaling (SOCS) and the protein inhibitor of activation of stat (PIAS) genes (Fig. 3 and Table 5). Table 5 shows that SOCS1 and SOCS3 were both upregulated in the TO-VS-IFN/SAV3 while SOCS3 was downregulated and SOCS1 was upregulated in the TO-VS-SAV3. Figure 5A and B shows network pathways linking the SOCS to other genes in the Jak/stat pathway in which all SOCSs were upregulated in the TO-VS-IFN/SAV3 (Fig. 5A), which conforms to observations shown in Table 4. The network pathway in the TO-VS- SAV3 shows a mixed representation of upregulated and downregulated SOCSs (Fig. 5B), which conforms to observations shown in Table 5 where SOCS1 was upregulated while SOCS3 was downregulated in the TO-VS-SAV3. As for PIAS, it was downregulated in the TO-VS-SAV3 and upregulated in the TO-VS-IFN/SAV3 (Fig. 5A and B).

### Apoptosis and cell proliferation genes

Genes that regulate apoptosis and cell-cycle activities were broadly classified into the Stat, MAPK and PI3K-AKT pathways as shown in Table 6 based on network pathways shown in Fig. 5A and B. In the Stat-pathway, the IFN regulatory factor 9 (IRF), creb binding protein (CBP) and threonine-protein kinase pim-1 (Pim-1) were upregulated in the TO-VS-IFN/SAV3 while in the TO-VS-SAV3, the signal transducer and adaptor molecule 2 (STAM2), CBP, Pim-1 and Pim-3 were downregulated (Table 6). In the MAPK pathway shown in Fig. 5A and B, the transcriptional regulator Myc-2 (Myc-2) and cyclin D (CycD1) were upregulated in the TO-VS-IFN/SAV3 while the Src homology region 2 domain-containing phosphatase-2 (SHP2) which is represented by the protein tyrosine phosphatase non-receptor 11 (PTPN11) in Table 5, son of sevenless (SOS), Myc-2, CycD, B-cell lymphoma extra-large (BclXL) and Sprouty-related EVH1 domain-containing protein 1 (S-pred) were all downregulated in the TO-VS-SAV3 (Table 6 and Fig. 5B). The phosphatidylinositol-4,5-bisphosphate 3-kinase (PI3K) and V-akt viral oncogene 2-like protein (AKT2) that regulate the PI3K-AKT-pathway for apoptosis were downregulated in TO-VS-SAV3 (Table 6) while only PI3K was downregulated in the TO-VS-IFN/SAV3. Network pathways linking the apoptosis and cell cycle genes for the TO-VS-SAV3 and TO-VS-IFN/SAV3 are shown in Fig. 5A and B. In general, all apoptosis and cell proliferation genes differentially expressed in the TO-VS-SAV3 were downregulated while the majority differentially expressed in the TO-VS-IFN/SAV3 was upregulated indicating that in the absence of type I IFN treatment SAV3 suppressed the expression of most of anti-apoptosis and cell proliferation genes.

**Table 6:** Apoptosis and cell proliferation target genes

Gene name	Abbr	Unigenes	NCBI	KEGG	TO-VS-SAV3			TO-VS-IFN/SAV3		
					Log2 (Fold change)	Reg	*P*-value	Log2 (Fold change)	Reg	*P*-value
A: Stat pathway genes										
Signal transducing adaptor molecule 2	STAM2	CL86.2	NP_001007370.1	K04705	-1.51	Down	1.76e-12			
Interferon regulatory factor 9	IRF9	CL11337.2	NP_001167190.1	K04693				1.65	Up	1.26e-13
CREB binding protein	CBP	CL7696.2	AAY85296.1	K04498	-2.67	Down	1.14e-06			
Threonine-protein kinase pim-1-like	Pim-1	CL9459.2	XP_003449193.1	K04702	-1.11	Down	2.38e-55	1.07	Up	5.80e-90
Threonine-protein kinase pim-3-like	Pim-3	CL1163.1	XP_003445092.1	K08807	-1.63	Down	9.75e-179			
B: MAPK Signaling pathway										
Protein tyrosine phosphatase, non-receptor 11	PTPN11	Unig19725	XP_003965393.1	K07293	-2.25	Down	2.21e-3			
Son of sevenless homolog 1	SOS1	CL9403.1	XP_003216158.1	K03099	-2.66	Down	3.28e-10	-1.06	Down	1.17e-3
Son of sevenless homolog 2	SOS2	Unig28623	XP_003443018.1	K03099	-2.57	Down	5.88e-07			
Transcriptional regulator Myc-2	Myc-2	Unig13199	NP_001167287.1	K04377	-2.16	Down	3.244e-07	1.11	Up	1.03e-05
G1/S-specific cyclin-D1	CycD1	CL11123.1	NP_001158863.1	K04503	-1.33	Down	3.33e-05			
G1/S-specific cyclin-D2	CycD2	CL3895.1	ACO14329.1	K10151	-2.23	Down	1.35e-168			
Apoptosis regulator Bcl-X	BcIXL	CL2019.1	ACI68003.1	K04070	-1.30	Down	1.21e-38			
Sprouty-related, EVH1 domain containing	Sprouty	CL10970.1	XP_003456564.1	K04703	-1.84	Down	7.73e-62			
C: PI3K-AKT pathway										
Akt RAC-beta serine/threonine-protein kinase	AKT	CL4020.1	ACY30363.1	K04456	-1.59	Down	3.83e-16			
Phosphatidylinositol-4,5-bisphosphate 3-kinase	PIK3CA	Unig30697	NP_998471.1	K00922	-1.64	Down	2.0e-5	-1.21	Down	4.84e-3
D: Other genes										
E3 ubiquitin-protein ligase CBL-B	CBL-B	Unig24091	XP_001921961.3	K04707				1.80	Up	3.48e-07

### Validation of RNA-Seq data by qRT-PCR

**Fig. 6:**
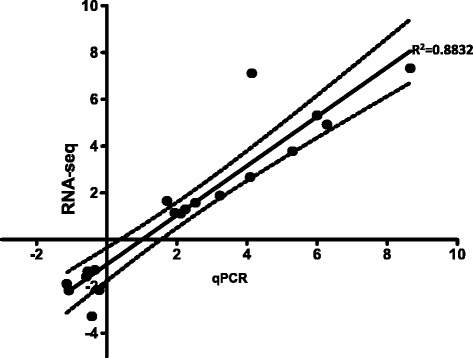
Shows a significantly high linear correlation (r^2^ = 0.8832, *P* < 0.0001) between RNA-seq and quantitative real time PCR (qRT-PCR) data for the TO-VS-IFN/SAV3 and TO-VS-SAV3. Dotted lines show the 95 % confidence limits for the correlation coefficient (r^2^) slope (95 % CI 0.8478 - 0.9771). qPCR data was generated as the mean of the fold increase in gene expression relative to the β-actin control group from three independent replicates (*N* = 3) of TO-cells simultaneously treated with type I IFN and infected with SAV3 (TO-VS-IFN/SAV3) together with none-IFN treated cells infected with SAV3 (TO-VS-SAV3). The cells used in this study were independent of cells used for RNA-seq data analysis

Figure 6 shows validation of RNA-seq data based correlation coefficient test between RNA-seq and qRT-PCR data. Our findings show a significantly high linear correlation (r^2^ = 0.8832, *P* < 0.0001) between RNA-seq and qRT-PCR for the TO-VS-IFN/SAV3 and TO-VS-SAV3. Put together, qRT-PCR confirms the validity of our RNA-seq data analysis by showing a high correlation between RNA-seq and qRT-PCR.

## Discussion

### *De novo* and reference gene guided transcriptome assembly

Current advances in next generation sequencing show that RNA-seq is poised to enhance our understanding of molecular networks of genes that form the innate and adaptive immune system in teleosts fish. In general, there are two approaches used to assemble a transcriptome from RNA-seq data which is by use of a *de novo* assembly or a reference gene guided approach. In situations where no reference gene exists as is the case for non-model organisms like Atlantic salmon, a *de novo* assembly is the only option for sequence assembly by which raw reads from RNA-seq are assembled into contigs without the guidance of a reference gene. Hence, in the present study we used a *de novo* approach to assemble a repertoire of genes that form the Jak/stat pathway from Atlantic salmon headkidney/macrophage like TO-cells with an aim to understand SAV3 modulation of responses post infection. Unlike microarray that uses predefined probes, *de novo* assembly provides a unique opportunity to identify novel genes which could be missed by none-coded micro-RNA probes. After *de novo* assembly, we used the TGICL software, which is a highly refined protocol used for the analysis of genes and EST sequences needed for transcriptome analysis [20]. Using this software, sequences are first cleaned to remove adaptors, low quality sequences without quality score, vector and adaptor contaminants and redundancies. Thereafter, sequences are searched pairwise and grouped into clusters that are assembled at high stringency to produce tentative concesus (TC) sequences. And as pointed out by Pertea et al. [19], individual assembly of each cluster to produce TCs has the advantage of producing large, more concensus sequences while eliminating potentially misclustered sequences. The TCs produced by the TGICL software have been used to construct species specific databases and to identify novel genes [20]. For example, several genes such as STAM2, SOS, and PIAS not previously reported from Atlantic salmon were matched to their orthologues in model species such as zebrafish and mammalia in this study suggesting that the Jak/stat pathway induced in macrophage/dendritic cell like TO-cells derived from Atlantic salmon headkidney cells expresses a repertoire of genes comparable to the Jak/stat signaling pathway observed in higher vertebrate species. On the other hand, we used a reference gene guided approach to detect and quantify the E2 SP of SAV3 based on the fact that the genomic sequence of SAV3 has been fully sequenced and characterized in salmonids [17, 28, 29]. Hence, both the *de novo* and reference gene guided assemblies were used in this study.

### Virus quantification in IFN I treated and non-treated TO-cells infected with SAV3

Virus replication in TO-cells of simultaneous IFN I/SAV3 treatment/infection (IFN/SAV3) was two logs (10^2^) lower than levels detected in SAV3-only infected cells. For the latter, it shows that SAV3 is a potent inducer of IFN signaling pathways in TO-cells post infection and also that the virus had the capacity to replicate in the presence of high levels of type I IFNs and timing of IFN I treatment is crucial [18]. These findings are consistent with observations made for other alphavirus studies in which it has been shown that timing in type I IFN treatment has a significant influence on replication of alphaviruses in infected cells [1, 30, 31].

### KEGG pathway analysis

RNA-seq is perfect for gene network construction and pathway analysis [32]. And as pointed out by Khatri et al. [33], pathway analysis has become the first choice to understand the underlying biological mechanisms of DEGs because it reduces the complexicity of RNA-seq data analysis whose vast array of sequence data is often difficult to interpret but instead combining RNA-seq with pathway analysis increases the explanatory power of transcriptome data analysis. In pathway analysis the first step is to generate a transcriptome of DEGs as shown in this study followed by searching for functional networks of genes expressed in the transcriptome based on the fact that there is a tendency for co-regulated genes to have functional correlations in the pathway. Khatri et al. [33] shown that pathway software development has progress through three different generations in the last decade. The first generation of pathway analytical softwares comprise of the over-representation analysis (ORA) softwares that include GenMAPP (http://www.genmapp.org), GOstat (http://gostat.wehi.edu.au) and GOToolBox (http://genome.crg.es/GOToolBox/). These software establish pathway analysis by putting together over expressed genes that show significant changes in expression based on gene level statistic [34, 35]. The second generation of softwares consist of functional class scoring (FCS) approaches such as the Gene Set Enrichment Analysis (GSEA) software (http://www.broadinstitute.org/gsea/) and GeneTrail (http://genetrail.bioinf.uni-sb.de) that compute both the statistical significance of individual genes that form a pathway (gene level statistic) and the statistical significance of the entire pathway (pathway level statistic) [36–38]. The third generation of softwares comprises of pathway topology (PT) based approaches such as the KEGG [39] and Panther DB [40] that do not only encorporate individual gene and pathway level statistics computed by the the ORA and FCS softwares, but they also provide information about gene products that interact with each other in a given pathway, how they interact (e.g. activation, inhibition, etc), and where thay interact (e.g. cytoplasm, nucleus, endoplasmic reticulum, etc). Hence, in this study we used the KEGG pathway analysis software which is a third generation PT based software to identify networks of genes that form the Jak/stat signaling pathway from a *de novo* assembled transcriptome with the aim to better understand the modulation of genes expressed by TO cells infected with SAV3. Consistent with all TP-based softwares, the KEGG pathways used in this study shows gene level statistical analyses for all DEGs and in terms of pathway level statistic analysis, our findings show that concurrent treatment of TO-cells with type I IFN together with SAV3 infection significantly enriched the Jak/stat pathway from a Qvalue of 5.889E-01 (P-value = 8.07E-01) to 1.136E-06 (P-value = 8.07E-08). This was supported by upregulation of several activator and signal transducer genes in the TO-VS-IFN/SAV3 group compared to the TO-VS-SAV3 group. As for genes that interact with each other and their interaction sites, the modulation is complex and below we pinpoint some of the key areas in the pathway where SAV3 infection interferes.

### Receptor genes that activate Jak/stat signaling

Type I receptor genes generated in response to SAV3 infection and type I IFN treatment were classified into the γC, gp130 and homodimer receptor families based on receptor classification used for higher vertebrates [41, 42]. Wang et al. [43] reviewed the γC receptor system for teleost fish in which they reported of IL-2RB, IL-4RA, IL-13RA and IL-15RA in salmonids which were also differentially expressed in this study. SAV3 infected cells (TO-VS-SAV3) had more type I receptor genes downregulated than type I IFN treated cells (TO-SV-IFN/SAV3) suggesting that SAV3 like other alphaviruses in higher vertebrates, inhibits the activation of several type I receptor genes post infection. The type II receptor family generated in this study was classfied into IL and IFN receptor genes. As for genes of the IFN receptor genes, IFNR1 and IFNR2 were expressed in both the TO-VS-IFN/SAV3 and TO-VS-SAV3 suggesting that activation of IFN-α/β receptors occurs as consequence of infection as well as IFN I treatment (as would be expected). However, it is important to point out that although our findings show the presence of both IFNR1 and IFNR2, which is in agreement with observations made by Ali et al. [44] who reported the presence of both IFNR1 and IFNR2 based on a transcriptome analysis of rainbow trout spleen. Studies carried out by Sun et al. [45] suggest that Atlantic salmon possess two clusters type I IFN receptor genes located on different chromosomes which allow for a large repertoire of IFN receptors than mammals and zebrafish. Hence, there is need for further studies to compare the IFNR1 and IFNR2 genes detected in this study with the IFN receptor genes repertoire reported by Sun et al. [45] and to determine their functional roles in the Jakstat pathway signaling. The expression of IFNGR1 and IFNGR2 was only found in the TO-VS-IFN/SAV3 cells. This suggests that SAV3 infection does not activate the IFNγ-receptors. The importance of IFN-γ in eliciting an antiviral effect in vitro remains non-conclusive. We have shown that IFN-γ exhibits marginal antiviral effect against SAV3 in vitro [16]. In contrast Sun et al. [46] suggested that recombinant trout IFN-γ had antiviral effect, although they also concluded that the observed effect of IFN-γ is partly dependent on IFN-α induction [47]. Additional studies are needed to understand the importance of IFN-γ in relation to SAV3 infection.

### Janus kinase and tyrosine phosphatase kinase genes

Four Jak-family members have been identified namely Jak1–Jak3 and Tyk2 in mammalia [48] and fish [49–52] of which Jak1, Jak2 and Tyk2 transcripts generated in this study were matched to their mammalian and fish orthologues. The mechanisms underlying activation, regulation and pleiotropic signaling in fish have not been studied in detail. Here we find that Jak1 was upregulated in SAV3 infected TO cells, with or without IFN-I treatment, while Jak2 and Tyk2 were only differentially expressed in the TO-VS-SAV3 groups, and importantly, they were both downregulated. Thus, these findings suggest that SAV3 infection activates Jak1 signaling while it suppresses activation of Tyk2 and Jak2. These observations are in line with what is seen for alphavirus infections in higher vertebrates in which blockage of Tyk2 was shown for Japanese encephalitis virus (JEV) [5], while Dengue virus type 2 was found to antagonize the Jak/stat pathway by downregulating Tyk2-stat signaling in human dendritic cells [53]. Lin et al. [5] showed that JEV infection blocked tyrosine phosphorylation of IFN associated Tyk2 without affecting the expression of IFN-α/β on the cell surface. We found that IFNα was upregulated when Tyk2 was downregulated, and while it is possible that SAV3 uses similar mechanisms to what is seen for other alphaviruses to block the phosphorylation of Tyk2 thereby inhibiting Jak/stat signaling in TO-cells, this remains to be shown.

### Signal transducer and activator of transcription genes

Type I IFN mediates tyrosine phosphorylation by binding to receptors associated with Jak1 and Tyk2 which activates Stats 1 and 2, while IFNγ mediates its signaling through Jaks 1 and 2 to activate Stat1 in mammalian cells [27, 42]. The TO-SV-IFN/SAV3 showed upregulation of IFNγ receptor genes, corresponding to the observed upregulation of Jak1 and Stat1, while downregulation of Tyk2 in SAV3 infected cells showed a corresponding downregulation of Stat3 and Stat5. Hence, it is possible that mechanisms in salmon and human cells are comparable, however, to identify the different Jaks that activate specific Stats in fish cells, there is need for detailed investigations.

Studies in higher vertebrates show that alphaviruses block the Jak/stat signaling pathway by inhibiting the phosphorylation of different Stats to prevent the transcription of antiviral genes in infected cells. For example, JEV infections block tyrosine phosporylation of Stats 1, 2 and 3 [46] while CHIKV nsPs were potent inhibitors of IFN-induced Jak/stat signaling by blocking stat1 phosphorylation of the Jak-receptors either by type I or II IFNs [1]. The observed downregulation of several type I and II receptor genes in SAV3 infected cells could resulted in inhibition of Jak2 and Tyk2 phosphorylation, which would align with the downregulation of most Stat genes. Although these data suggest that SAV3 could be using mechanisms similar to those used by mammalian alphaviruses to block the Jak/stat pathway, there is need for detailed investigations to consolidate these observations in TO-cells.

### Negative regulatory genes

The negative regulatory genes linked to inhibition of the Jak/STAT pathway include genes that form a negative feedback loop comprising of members of the SOCS family and genes that directly inhibit the Jak/stat pathway such as PTP1b, SHP and PIAS. In salmonids, several SOCS genes have been cloned and characterized [53, 54] and studies carried out by Skjesol et al. [55] show that SOCS1 supresseses type I and II signaling of the Jak/stat pathway in Atlantic salmon. In mammalia, SOCS1 and SOCS3 but not SOCS2 inhibit IFN-mediated antiviral activities [52]. In this study, SOCS1 was upregulated in both TO-VS-IFN/SAV3 and TO-VS-SAV3 while SOCS3 was only upregulated in the TO-VS-IFN/SAV3 and downregulated in the TO-VS-SAV3 suggesting that SAV3 replication could have suppressed the expression of SOCS3.

Among the genes that directly inhibit Jak signaling, only PIAS was differentially expressed and it was upregulated in the TO-VS-IFN/SAV3 and downregulated in the TO-VS-SAV3, suggesting that SAV3 suppressed the expression of PIAS in TO-cells. Although PIAS has not been characterized in salmonids, its expression at transcript level in this study suggests that it exists in Atlantic salmon and that it likely to play an important role in regulating the Jak/stat pathway in TO-cells. Overall, these negative regulators were all downregulated in the TO-VS-SAV3 cells, except for SOCS1, suggesting that SAV-3 upregulates SOCS1 to prolong its survival in infected cells.

### Apoptosis and cell-proliferation genes

Here we find that apoptosis related genes Pim-1, c-Myc and CycD are upregulated in the TO-VS-IFN/SAV3 and downregulated in the TO-VS-SAV3 cells. Apoptosis following virus infection is an important defense mechanisms given that cell autodestruction is one of the safest ways of limiting virus spread to neighboring cells in an infected host [56]. However, viruses have evolved defense mechanisms that evade or delay apoptosis [56–58] by altering the expression of anti-apoptosis target genes that regulate cell growth and proliferation [59–62]. Our finding can be interpreted as a survival strategy of SAV3 to prolong its replication cycle in TO-cells. Further, it is also possible that the expression of PI3K and AKT could be regulating SAV3 induced apoptosis in TO-cells as shown for other alphavirus where PI3K-AKT pathway regulates cell survival by preventing caspase-8 activation to prevent apoptosis in virus infected cells [63–65]. Mastrangelo et al. [66, 67] have shown that Bcl-2 and BcI-xL limit apoptosis upon infection with alphaviruses while CycD has been shown to exert the same effects in neuronal cells infected by Sindbis virus [68]. Similarly, Pim-1 acts synergestically with c-Myc [61], to inhibit apoptosis related mitochondrial dysfunction through the bcl-2 dependent pathway [62, 69]. It remains to be shown whether downregulation of apoptosis target genes in the TO-VS-SAV3 cells could be an evasion strategy to shutdown cell-death or to slowdown apoptosis to prolong replication time in infected TO-cells.

## Conclusion

This study provides a global overview of genes that form the Jak/stat pathway induced by SAV3 infection and the combined IFN I/SAV3 infection allow us to pinpoint modulating effects of SAV3 infection on the Jak/stat signaling pathway. It is important to note that the data presented here only provides a repertoire of downregulated and upregulated genes interlinked in a *de novo* assembled network pathway analysis, and does not show the exact mechanism by which the modulatory processes take place. Thus a lot of work remains to be done to elucidate the exact interaction points and modulation of the Jak/stat encoded proteins. genes generated by *de novo* assembly in this study. Further, the study shows that combining *de novo* assembly with pathway based analysis increases the explanatory power of transcriptome data analysis by putting together a network of genes that regulate host-pathogen interaction processes at cellular level. We anticipate that the Jak/stat pathway genes put forth in this study shall serve as a roadmap for elucidating molecular mechanisms used by SAV3 infection to evade host defensins at cellular level.

## Data access

The RNA-sequencing data generated in this study has been deposited in the National Center for Biotechnology Information (NCBI) Gene Expression Omnibus (GEO) database accession number GSE64095 (www.ncbi.nih.gov/geo Accession number GSE64095).
